# Quality of work-life and turnover intentions among the Ghanaian nursing workforce: A multicentre study

**DOI:** 10.1371/journal.pone.0272597

**Published:** 2022-09-01

**Authors:** Collins Atta Poku, John Ndebugri Alem, Rasheed Ofosu Poku, Sandra Adwubi Osei, Edward Obeng Amoah, Adelaide Maria Ansah Ofei

**Affiliations:** 1 Department of Nursing, Kwame Nkrumah University of Science and Technology, Kumasi, Ghana; 2 School of Nursing and Midwifery, University of Ghana, Legon-Accra, Ghana; 3 School of Nursing and Midwifery, University for Development Studies, Tamale, Ghana; 4 Directorate of Family Medicine, Komfo Anokye Teaching Hospital, Kumasi, Ghana; 5 Department of Nursing, Seventh Day Adventist Hospital, Kwadaso–Kumasi, Ghana; 6 Seventh Day Adventist Nursing and Midwifery Training College, Kwadaso-Kumasi, Ghana; Jacobs University Bremen, GERMANY

## Abstract

**Introduction:**

Attrition of the Nursing Workforce from low-and middle-income countries to high-resourced settings is a reality that has escalated in the current Coronavirus pandemic due to varied reasons. With increased job stress resulting from the pandemic, the Quality of Work-Life of the Nursing Workforce is affected, with its effect on poor quality care to the client. This study sought to assess the perception of the Nursing Workforce about the Quality of Work-Life, and the factors that predict turnover intention among nurses in the Kumasi Metropolis, Ghana.

**Methods:**

A cross-sectional, descriptive design involving multiple centres was used. The participants were made up of 348 Registered Nurses working in primary, secondary, and tertiary healthcare in five (5) hospitals in the Kumasi Metropolis. Data collection was done using questionnaires adapted from the Work-Related Quality of Life Scale and the Turnover Intention Scale and analyzed using frequencies, mean, standard deviation, Pearson’s Product Moment Correlation, and Multiple Regression.

**Results:**

The Registered Nurses perceived Quality of Work-Life as low; with close to half of them having a turnover intention. All the domains of Quality of Work-Life of the Nursing Workforce significantly correlated with Turnover intentions. Regression analysis showed that the number of years in a healthcare setting, general well-being, job control and satisfaction, and working condition of the Registered Nurse significantly predicted their turnover intentions at the p-value of 0.05.

**Conclusion:**

The findings of the study have provided an understanding of the Quality of Work-Life, and factors that contribute to increased turnover intentions among the Nursing Workforce amid the COVID-19 pandemic. Healthcare systems must enrol in requisite programmes that provide psychological and social support through counselling to promote the Quality of Work-Life of nurses.

## Introduction

Globally, the Coronavirus disease 2019 (COVID-19) pandemic has created an enormous danger to the public, particularly the health workforce due to its high virulence, coupled with the anxiety and psychosocial stress associated with the sequel of the disease. With the increased number of daily infections, most Registered Nurses (RNs) experience anxiety and burnout (emotional exhaustion and depersonalization) [1–3]. If unresolved, these challenges can adversely impact the Quality-of-Work-Life (QoWL) of RNs. Though a negative association between COVID-19 related stress and QoWL of the RNs can be expected [4, 5], there has not been any data to formally ascertain the proposition in the context of sub-Saharan Africa (SSA).

QoWL is a multidimensional concept that defines the employee’s feelings about a variety of aspects of one’s work; thus, job content, duty discretion, work environment, adequate and fair remuneration, career prospects, work-life balance, shared decision-making, health and safety, burnout, and job security [3, 6]. It almost manifests itself in the dynamic relationship between the workforce and the overall external practice environment. QoWL is affected by several factors, both intrinsic and extrinsic and it is considered an important phenomenon in organizational effectiveness which can affect workers at one time or another if little attention is given to it. Anger, frustration, and burnout are common among workers and can be costly for the staff, patients, and organizations alike [3, 4].

Before COVID-19, various studies found low to moderate levels of QoWL in RNs, with demographic characteristics of RNs such as age, gender, marital status, education, position and location of the job, hospital type, salary, and work experience being significant contributory factors, thus, these variables can be of importance in developing programmes to enhance the QoWL of RNs [4–6]. A study in Saudi Arabia found over 50% of nurses to be dissatisfied with the QoWL [7], while a similar study among Iranian nurses, reported a very higher proportion (70.8%) of participants having a low QoWL [8]. However, in High-Resourced Countries, it is been observed that two-thirds of RNs have very high levels of QoWL and this has been attributed to a safe and healthy practice environment, nursing leadership support, an opportunity for professional/career development, adequacy of resources, and measured workload [8, 9].

QoWL when given adequate attention, contributes positively to the realization of organizational goals. Thus, programmes that allow workers to manage their work and out-of-work lives increase productivity [10]. Moreover, the recognition and support by the organization through its stated values and policies of the obligations can relieve the external stresses of employees. This allows them to concentrate on their jobs during the working day and helps to reduce absenteeism. The effect can both improve productivity and increase employee engagement and loyalty [11].

The ability to show empathy (an innate understanding of the pain and suffering of others)–in providing care to patients, which is inherent in the role of RNs, is also linked to their QoWL. It has been suggested that when RNs lose the balance between emotions, thoughts, reactions, and interpersonal dynamics, empathy is replaced by sympathy, and hope by despair. In this context, RNs tend to be little concerned about their individual or organizational effectiveness but are affected more by the events around them [10–12]. Thus, poor QoWL of RNs has a significant negative effect not only on their functioning and the care received by patients but has a detrimental effect on the effectiveness of the hospital in providing intended high-quality healthcare.

A study in Ghana pre-COVID 19 showed a sense of fulfilment among RNs in some work factors affecting their QoWL [13], Darteh-Baah likewise found positive manager-subordinate relationships to be another key factor determining the QoWL of staff [14]. The job of RNs in low- and middle-income countries (LMICs) like Ghana is associated with the enormous stress of providing high-quality patient care with very limited human and material resources, especially in the COVID-19 pandemic. These high demands from patients result in increased workloads and stressful non-standard work schedules [13, 15].

To resolve the challenges of poor QoWL of RNs, a broad range of issues which include workload, managers’ leadership and clinical support, appropriate continuing professional education, employee safety, and satisfactory remuneration has been suggested and needs to be addressed holistically [16].

In addition to the challenges posed by COVID-19 on QoWL of RNs, researchers have demonstrated that there is an increased turnover intention among RNs in high-resourced countries due to the high rate of COVID-19 associated morbidity and mortality among the health workforce in these settings [4–6]. The combined effects of shortage and exodus of the nursing workforce is a challenge globally but it is more prominent in LMICs, and this has contributed to an uneven distribution of RNs across different care settings. Previous studies have identified many factors contributing to RNs’ turnover intention, and these may vary between RNs in hospitals in different geographical settings [17]. In a study by Potira et al. [18], pre-COVID-19, more than 60% of RNs expressed their intention to leave the nursing profession, and it was attributed to poor remuneration, lack or inadequate work motivations, and lack of career improvement opportunities [19]. These factors coupled with reduced job satisfaction account for the low QoWL of the RNs.

Improved QoWL reduces employee turnover rate, thus saving the organizational costs of recruitment and training of new workforces. Given the benefits of ensuring positive QoWL of staff, many health organizations include the consistency of workers’ work experience in the evaluation of the hospital. Similarly, socially responsible corporate bodies, pay particular attention to QoWL when making investment decisions [7–9]. It has been reiterated by Blaauw et al. [16] that, QoWL should be considered a vital element in the staff recruitment and retention cycle, which has a significant influence on retaining the more skilled and experienced nursing workforce in work settings.

The prevailing situation in Ghana’s healthcare system as influenced by COVID-19 is not different from the global picture. The circumstance is characterized by an inadequate nursing workforce, stressful work demands and schedules, and limited material resources to facilitate work; and these factors lead to the poor QoWL of RNs. On improving the situation of the healthcare system in providing standard care to patients, efforts must be made to understand the various factors negatively impacting the QoWL of RNs and develop appropriate solutions to address them [7, 17, 20]. Focusing on the overall QoWL of staff by safeguarding their general welfare in the organization will lead to positive nursing jobs- and patient outcomes. Universally, amidst COVID-19, there is poor QoWL and various predictors have also been associated with the turnover of RNs [21, 22]; there is, however, a paucity of research in Ghana which explains the QoWL among RNs in public healthcare settings relative to turnover intention. Hence, the study aimed to find out RNs’ perceptions about their QoWL and the influence of QoWL on turnover intention among RNs working in public healthcare facilities in the Kumasi Metropolis.

### Theoretical framework

The study is informed by the Job Demands-Resources (JD-R) model by Bakker and Demerouti [23]. The model centres on organizational or occupational health and explains the negative and positive aspects of well-being. It is hypothesised that, for any organization, there are specific job demands and job resources, and their interaction determines either positive or negative outcomes [23, 24]. Job demands are characteristics of the work environment, that require continuous physical and psychological efforts, and may result in undesirable outcomes. Job resources, conversely, reduce the job demands and catalyses one’s growth and quality of life in an organization. The JD-R model operates on the assumption that, for any work environment, job demands may induce negative outcomes including stress, burnout, job dissatisfaction, increased turnover, etc whereas job resources bring about higher QoWL, increased work engagement, and productivity [25]. As applicable to this study, the COVID-19 pandemic has led to negative impacts on nurses’ QoWL and their general well-being [26]. The individual who may not be able to cope well with the impact may end up quitting the job. Studies have found a relationship between QoWL and turnover intention [27–29].

### Hypothesis

H_1_: There is a negative relationship between the QoWL and the turnover intentions of nurses.

### Operational definitions of the dimensions of WRQoL

**General Well-Being (GWB)** reflects both the psychological and general well-being of nurses. The work environment and GWB influences each other. Good GWB of nurses promote the QoWL of the nurse.

**Home-Work Interface (HWI)** measures the degree to which the individual acknowledges their employer’s appreciation and attempts to support them by relieving the stress external to work. The sub-scale is related to work-life balance; thus, control over work. HWI is accomplished when there is a mutual benefit between the individual and the work or organization.

**Job and Career Satisfaction (JCS)** is the degree to which employees are content with the work and its prospects. It explains how one feels how the work environment offers them fulfilment, high self-esteem, etc. The JCS of the nurse is influenced by factors such as role ambiguity, reward system, career development and prospects, remuneration etc.

**Control at Work (CAW)** depicts the rate at which nurses feel they are engaged in decision making that affects them in an organization. The CAW shows the degree to which nurses can exercise an appropriate level of control within their work environment.

**Working Conditions (WCS)** measures the rate at which nurses feel satisfied with the conditions in the work environment—resources, safety, leadership, working hours and shift patterns, salary level etc necessary for the work

**Stress at Work (SAW)** rates the extent of pressures and demands associated with nurses’ work. The work pressures and demands can be a positive aspect of an organization, but when it is excessive and beyond the coping abilities of the individuals, stress can ensue.

## Materials and methods

### Study design and setting

The study employed a multicentre cross-sectional approach at public healthcare facilities in Kumasi Metropolis, Ghana from 22^nd^ September 2020 to 30^th^ November 2020. There were 46,062 confirmed cases of COVID-19 with 297 deaths when the survey started, and 51,667 confirmed cases with 323 deaths when the survey was ended. According to Ghana Health Service, 3580 health workers contracted COVID-19 [30]. The Kumasi metropolis has the highest number of health facilities in the Ashanti region at 38%. Five (5) hospitals were chosen for the study including a tertiary facility, 3 secondary government facilities and one faith-based facility. These facilities were chosen due to their significant role during the COVID-19 pandemic as treatment centres and their wide accessibility and usage by residents in Kumasi and other parts of Ghana and overseas. Strengthening the Reporting of Observational Studies in Epidemiology (STROBE) was used as a reporting guideline for the study [31].

### Participants

The participants were RNs with clinical roles at the five (5) hospitals in the Kumasi metropolis. Eligibility criteria for the study included RNs who have worked for at least one year in the current post. This is justified in the sense that they have practised in the work environment and have sufficient experience to be able to evaluate their QoWL during the COVID-19 pandemic. RNs who were on leave and/or those who did not have any direct role in the care of patients who reported to the selected healthcare facilities were excluded from the study.

### Sample and sampling technique

The total number of RNs in the Ministry of Health through its implementing agencies in Kumasi is 4243 (GHS Annual Report, 2018). Using a confidence level of 95% and 0.05 alpha level, the sample size for RNs was computed using Slovin’s simplified sample size formula [32], and the computed sample size was 366. Considering non-responsiveness, missing or accidental error of the samples, the sample was adjusted by 10% to 403. Proportionate stratified sampling was used to select the RNs based on the 5 facilities’ numerical strength. A convenient sampling technique was used to sample participants for the study.

### Measures

Two instruments were used for the data collection. Section A was on the demographic profile and work-related data of the participants including gender, age, number of years in nursing, marital status, and salary level. Section B was a validated *“The Work-Related Quality of Life Scale (WRQoL)”* which is a measure of QoWL [33]. The WRQoL measure is having a total of 23 items with six subscales. The subscales include the General Well-Being dimension (6 items), Home-Work Interface (3 items), Job and Career Satisfaction subscale (6 items), Control at Work subscale (3 items), Working Conditions subscale (3 items), and Stress at Work subscale (2 items). The tool is a 5-point Likert scale with 1 denoting strongly disagree and 5 denoting strongly agree. The average of the number of test items was used to estimate the score of individual sub-scales; while high scores indicated higher QoWL, low scores showed a lower QOWL. Three (3) of the test items (7, 9, and 19) were reversely scored. The tool is validated globally [34], and all the studies that have used it have reported an acceptable Cronbach’s alpha value between 0.88 and 0.95 [34–40].

The outcome variable, the turnover intention was measured using three items from Kelloway et al.’s *Turnover Intention Scale* [41]. These items were rated on a Likert scale ranging from strongly disagree (1) to strongly agree (5). A higher score above average indicated a higher turnover intention. The tool has an acceptable Cronbach alpha of 0.92 [42].

### Data collection

The participants were contacted in person to enlist their participation. Informed consent was given by participants who expressed the willingness to be part of the study. Self-administered questionnaires were given to participants for completion.

### Data analysis

The data were analyzed with the SPSS version 25 software programme. The descriptive statistics included the percentage, frequencies, mean and standard deviations. In a simple bivariate analysis, the Pearson’s Product Moment Correlation (Pearson *r*) was used to determine the strength and significance association between the domains of QoWL and turnover intentions of the RNs, while hierarchical regression analysis (enter method) was conducted on the predictors of turnover intention. The significant variables of the RNs’ age, number of years at the present post, salary levels, living with a spouse, and type of shift were adjusted in the first model. The QoWL variables (general wellbeing, home and work interface, control at work, working conditions, job career satisfaction, and stress at work), were adjusted in the second model. The normality of the data was checked using the Kolmogorov Smirnov test. Homoscedasticity, singularity and multicollinearity were also checked to satisfy the assumptions of regression. The data analysis was conducted at a statistical significance of *p* < 0.05.

### Ethical consideration

Ethical approval (KATH IRB/AP/094/20) was sought from the Institutional Review Board of Komfo Anokye Teaching Hospital (KATH-IRB) and approval from the management of the facilities before the commencement of the research. Following acceptance to participate in the study, participants gave verbal consent. Participants were assured of confidentiality, and that any expressions that may serve to lead to their identification will not be included. They were also informed about their right to withdraw their participation at any point in the study with no associated effect on them.

## Results

### Sociodemographic characteristics of participants

With a total of 403 distributed surveys, 360 were returned. Twelve (12) returned questionnaires were, however, removed from the data analyses as seven were having a lot of missing data and the other five did not meet the inclusion criteria of working in the current post for at least 1 year. The response rate was 86.4% (348/403).

The mean age of participants was 34±4.73 years. As shown in Table 1; most of the study participants (81.6%, n = 284) were females. Participants with a diploma as their highest qualification were the majority (54.0%, n = 188). Out of the 211 (60.6%) participants who were married, 126 (59.7%) lived with their spouses. Approximately 47% (162) of participants had worked between 5 and 10 years at the current post. Most of the participants (196, 56.3%) worked on an 8-hour shift system and 182 (52.3%) earned at most Ghc2,500.00 (US$450) as a monthly salary.

**Table 1:** Sociodemographic and job characteristics of participants in the study.

Variable	Category	Frequency (N = 348)	Percentage
Gender	Male	64	18.4
	**Female**	**284**	**81.6**
Marital status	Single	129	37.1
	**Married**	**211**	**60.6**
	Separated	5	1.4
	Widowed	3	.9
Living with spouse	**Yes**	**126**	**59.7**
	No	85	40.3
Highest qualification	**Diploma**	**188**	**54.0**
	Bachelor’s	96	27.6
	Master’s	64	18.4
Number of Years at the current post	1–4	144	41.4
	**5–10**	**162**	**46.6**
Salary level (US$)	Above 10	42	12.0
	**Below US$450.00**	**182**	**52.3**
	US$450.00–600.00	137	39.4
Types of shifts	Over US$600.00	29	8.3
	**6 to 8 hours**	**196**	**56.3**
	More than 8 hours	152	43.7

Field Data (2020).

### Perceived QoWL among registered nurses

The findings of the RNs’ perception of their QoWL are presented in Table 2. The mean score for overall QoWL (71.64) signifying a percentile equivalent of 62; implying that the QoWL among RNs is low. A greater proportion of participants (69.5%, n = 242) rated their overall QoWL as low. In terms of the domains of the QoWL scale; majority of RNs had low QoWL on general wellbeing (64.4%), home and work interface (63.8%), control at work (69.8%), working conditions (82.5%), and stress at work (67.2%). Again, the highest number of participants (45.1%) rated low QoWL on job career satisfaction.

**Table 2:** Level of QoWL among RNs.

QoWL domain	Level of quality	Frequency (N = 348)	Percentage	Mean	SD
General Wellbeing	**Low QoWL**	**224**	**64.4**		
	Average QoWL	30	8.6	19.15	3.781
	Higher QoWL	94	27.0		
Home Work Interface	**Low QoWL**	**222**	**63.8**		
	Average QoWL	77	22.1	8.68	2.659
	Higher QoWL	49	14.1		
Job Career Satisfaction	**Low QoWL**	**157**	**45.1**		
	Average QoWL	79	22.7	20.44	4.720
	Higher QoWL	112	32.2		
Control at Work	**Low QoWL**	**243**	**69.8**		
	Average QoWL	59	17.0	8.70	3.093
	Higher QoWL	46	13.2		
Working Conditions	**Low QoWL**	**287**	**82.5**		
	Average QoWL	22	6.3	8.20	2.791
	Higher QoWL	39	11.2		
Stress At Work	Low QoWL	78	22.4		
	Average QoWL	36	10.3	6.46	2.064
	**Higher QoWL**	**234**	**67.2**		
Overall QoWL	**Low QoWL**	**242**	**69.5**		
	Average QoWL	54	15.5	71.64	12.607
	Higher QoWL	52	14.9		

### Turnover intentions among RNs

Table 3 presents the turnover intentions among RNs. A greater proportion of participants (47.7%, n = 166) agreed that they plan on leaving their job within the next year. Additionally, 44.0% agreed that they do not want to remain in their job and 44.2% said they have been actively looking for other jobs.

**Table 3:** Turnover intention among RNs.

Turnover intention	Response	Frequency(N = 348)	Percent (%)	Mean	SD
I plan on leaving my job within the next year	Disagree	117	33.6		
	Neutral	65	19.0	3.13	1.296
	**Agree**	**166**	**47.7**		
I have been actively looking for other jobs	Disagree	138	39.7		
	Neutral	56	16.1	3.02	1.364
	**Agree**	**154**	**44.2**		
I do not want to remain in my job	Disagree	125	35.9		
	Neutral	70	20.1	3.16	1.439
	**Agree**	**153**	**44.0**		
Overall turnover intention				3.10	1.366

(Field data, 2020).

### Relationship between QoWL and turnover intention among nurses

To establish the relationship between the QoWL and turnover intention, the Pearson product-moment correlation analysis was conducted. As presented in Table 4, the results showed a statistically significant correlation between all the domains of QoWL and turnover intention. There was a significant negative correlation between overall QoWL and turnover intention (r = -.406, *p* < .001). This is a moderate correlation and implies that an increase in overall QoWL among nurses leads to a decrease in turnover intention. Similarly, a statistically significant negative correlation was found between general wellbeing (r = -.266, *p* < .001), homework interface (r = -.348, *p* < .001), job career satisfaction (r = -.358, *p* < .001), control at work (r = -.266, *p* < .001), working conditions (r = -.356, *p* < .001) and turnover intention. However, there was a significant but weak positive correlation between stress at work and turnover intention among nurses (r = .099, *p* < .001).

**Table 4:** Correlations among the QoWL dimensions and turnover intentions.

Variables	1	2	3	4	5	6	7	8
1. General Wellbeing	-							
2. Home Work Interface	.322**	-						
3. Job Career Satisfaction	.352**	.502**	-					
4. Control at Work	.224**	.480**	.660**	-				
5. Working Conditions	.294**	.529**	.468**	.388**	-			
6. Stress at Work	-.017	-.227**	-.019	-.001	-.078	-		
7. Overall QoWL	.623**	.693**	.848**	.747**	.679**	.097	-	
8. Turnover Intention	-.266**	-.348**	-.358**	-.266**	-.356**	.099*	-.406**	-

** = Correlation is significant at 0.01 level (2-tailed),

* = Correlation is significant at 0.05 level (2-tailed).

### Predictors of turnover intentions among RNs

The multiple linear regression results in Table 5 supported the model. The QoWL variables were entered into the model; and it explained 21.1% of the variation (R^2^ = 0.211, F(_6, 341_) = 15.220, *p<0*.*05*). The general well-being (β = -.121, p < 0.02), job control and satisfaction of the RN (β = -.179, p < .0.05), working conditions (β = -.173, p < 0.05) and stress at work (β = .116, p < 0.05) were significant predictors of turnover intention.

**Table 5:** Predictors of turnover intentions among RNs.

		B	SE	Β	T	Sig.
Model	(Constant)	15.754	1.188		13.262	.000
	**General Well Being**	**-.116**	**.051**	**-.121**	**-2.290**	**.023**
	Health Work Interface	-.133	.088	-.097	-1.516	.131
	**Job control and satisfaction**	**-.138**	**.054**	**-.179**	**-2.560**	**.011**
	Condition at Work	-.099	.078	-.007	-.110	.912
	**Working Conditions**	**-.226**	**.077**	**-.173**	**-2.924**	**.004**
	**Stress at Work**	**.204**	**.088**	**.116**	**2.314**	**.021**

Summary: R^2^ = 0.211, F(_6, 341_) = 15.220, p≤.001.

Dependent Variable: Turnover intentions 95% confidence level (α = .05).

## Discussion

Globally, the nursing workforce shortage was highlighted as a major challenge even before the COVID-19 pandemic. From literature and the findings of this study, the situation has worsened in the past year of the pandemic [5, 43]. This study was a self-perception survey that evaluated the QoWL of RNs and the predictors of turnover intention. The study findings established that RNs reported perceived poor QoWL and a high turnover intention in the COVID-19 era.

This finding aligns with the International Council of Nurses (ICN) report [5, 28, 43], which indicated a higher turnover intention of the nursing workforce globally, especially, during the COVID-19 pandemic. The cause has been associated with increased workloads, lack of or inadequate material resources, emotional exhaustion, and stress. Though few studies have been undertaken on the subject in many countries, some developed countries have established the rate of turnover and/ or turnover intentions. For instance, while the United Kingdom reported a 20% pre-pandemic and 36% pandemic nursing workforce turnover rate; Canada reported 29.5% and 22.3% high intention to leave the workplace and the nursing profession, respectively during the pandemic but 13% and 19.9% pre-pandemic turnover rate in previous studies [29–31].

Turnover rate and turnover intention are not only vastly influenced by job characteristics but also the personal features of the RN. The current study found that RNs’ increased number of years in the job setting is negatively related to turnover intention. This is in line with findings [44] which stated that RNs in Europe with job experience in the nursing profession are negatively related to turnover intention and/ or turnover. Meanwhile, job dissatisfaction resulting from poor practice environments (increased workloads, emotional exhaustion, and increased work-family interface) as magnified by the COVID-19 pandemic [45] has gravely contributed to turnover intention across many settings as mentioned also in the Philippines. For most settings, the reasons are always deduced from low QoWL with its associated increased job stress, poor well-being of the RN, and poor working conditions at the workplace. On the labour front, work satisfaction cannot be discussed without an interest in contingent rewards and salary levels. Though in the current study, no significant relationship was established between salary level and turnover intention, it is important to ascertain that in other jurisdictions, monetary incentives are key factors for predicting turnover among RNs [46]. This factor and others as elucidated in our findings coupled with the catastrophic nature of the pandemic-induced fear and increased job demands that escalate RNs’ turnover intentions.

Moreover, with RNs’ perceived poor QoWL, study results from other countries such as Iran [47], Saudi Arabia [48], Malaysia [49], and Australia [50] also revealed that RNs who cared for COVID-19 patients had poor working conditions and increased workload and as such low QoWL. A study in the United State, however, showed a moderate level of professional quality of life [51]; this could be attributed to the nature of the working environment and the adequacy of both human and material resources at their disposal in fighting the COVID-19 pandemic [52]. In sub-Saharan Africa, data on QoWL among RNs in the COVID-19 pandemic is scanty.

On average, most of the evidence points to concerns such as increased workloads resulting from the higher numbers of both COVID-19 and non-COVID-19 patients that reported to healthcare settings. Other challenges were COVID-19 precaution related, as they do not get adequate personal protective equipment (PPE) to work on the clients. The perceived higher stress level among RNs could be due to the disease burden and fear of contracting the infection and also the fear of infecting family members which ultimately impacted the RNs’ home and work interface [25–27]. This outcome affects RNs’ physical and emotional well-being, and underscore the need to improve the home and work interface, and working condition as well as reduce stress in the practice environment, and develop policies necessary to enhance job career satisfaction.

Though the present study did not emphasize the factors that may lessen the rate of turnover rate, other studies suggest adequate emergency preparedness of RNs in receiving both infected and potentially infected patients reporting to the healthcare facility, RNs’ autonomy, and the nurse managers’ leadership style as potential means to reduce the canker [55].

The data on QoWL and turnover intention of the RNs (both leaving the current work setting and/or the profession) is limited and still emerging in LMICs. Relying on the global data and the ones from a few specific settings will go a long way to improve the QoWL of the RNs thereby curtailing the menace of nursing job turnover in the already fragile health system brought by the COVID-19 pandemic in LMICs. Nurse leaders in policy formulation can play an imperative role through both short and long-term training, engagement and motivation strategies to influence the QoWL and the retention of RNs. Again, retaining experienced practitioners who can in turn provide peer support can be a key to reducing the turnover of nurses. Empowering RNs to contribute to job satisfaction and organizational commitment (nurse engagement), ultimately improves the challenge of increased turnover intention and turnover.

### Limitation

As in cross-sectional study designs, the data were collected at a specific period; the relationship between QoWL and the turnover intention can change as the impact of the pandemic intensifies. We cannot however establish the causal effect of QoWL of RNs on turnover intentions. Again, the use of a self-reported questionnaire might have a potential inherent bias. The sample had a relatively young group with a mean age of 34(±4) years, thus generalizations should be made with caution; the age composition of the sample is, however, largely reflective of the nursing workforce in Ghana. Furthermore, there was no baseline data on the QoWL of the nurse in the study setting before the COVID-19 pandemic, therefore comparing the QoWL of nurses pre-and the pandemic era was not possible. Future studies will consider a large-scale survey of the QoWL of nurses and their coping strategies from rural and urban settings in Ghana, as the present study focused on only urban centres. Again, employing a mixed-method research approach would have provided clarity and an in-depth understanding of the conditions that contribute to lower QoWL among Ghanaian nurses; and this could be considered for future studies. However, strategies adopted to ensure validity and reliability of the study rule out the above deficiencies, for instance, the sample size is adequate for a quantitative study and the research tools adjusted for the researchers’ context.

### Implication

The study found that the QoWL of nurses was low, and implies that it can have adverse effects on patient care, as a dissatisfied staff is associated with poor patient outcomes including a higher incidence of adverse event reporting [53, 54], workforce burnout, absenteeism, increased turnover and its associated economic implications for the organization [28, 55, 56]. Providing support systems such as facilitative supervision, effective leadership, supportive team, effective communication, reduced work-life conflicts, and shift flexibility [38, 57], would lessen the stress of RNs thereby improving their QoWL and reducing the rate of turnover among RNs in healthcare facilities.

## Conclusion

The value and contribution of the health workforce especially RNs in the COVID-19 pandemic has highlighted the necessity to improve the QoWL of RNs. With the increased intention of nurses leaving their profession or job coupled with the continuous threat of the COVID-19 pandemic, the morale and safety of RNs remain an important feature in ensuring quality care delivery. Given the goodwill from the public, nursing leaders should improve on the allocation of human and material resources to support the nursing profession recovering from the pandemic. The general impression of improving the general well-being of nurses and also the working condition is urgent to safeguard the QoWL of RNs and by extension to reduce turnover intention. As a human resource management strategy, it is important to increase the recruitment and retention of the health workforce through all means possible to reduce stress and burnout in the remaining nursing workforce.

## Supporting information

S1 Dataset (ZIP)

## Abbreviations

COVID-19: Coronavirus disease
GHS: Ghana Health Service
ICN: International Council of Nurses
IRB: Institutional Review Board
KATH: Komfo Anokye Teaching Hospital
LMICs: Low- and Middle-Income Countries
PPE: Personal Protective Equipment
QoWL: Quality of Work-Life
RNs: Registered Nurses
SDA: Seventh Day Adventist
SSA: sub-Saharan Africa
STROBE: Strengthening the Reporting of Observational Studies in Epidemiology
WRQoL: Work-Related Quality of Life
